# Prepulse inhibition in patients with bipolar disorder: a systematic review and meta-analysis

**DOI:** 10.1186/s12888-019-2271-8

**Published:** 2019-09-11

**Authors:** Zhen Mao, Qijing Bo, Weidi Li, Zhimin Wang, Xin Ma, Chuanyue Wang

**Affiliations:** 1 The National Clinical Research Center for Mental Disorders & Beijing Key Laboratory of Mental Disorders & Beijing Institute for Brain Disorders Center of Schizophrenia, Beijing Anding Hospital, Capital Medical University, No.5 Ankang Lane, Dewai Avenue, Xicheng District, Beijing, 100088 China; 2 Advanced Innovation Center for Human Brain Protection, Capital Medical University, Beijing, 100069 China

**Keywords:** Bipolar disorder, Healthy controls, Prepulse inhibition, Systematic review, Meta-analysis

## Abstract

**Background:**

Prepulse inhibition (PPI) is a measurement method for the sensory gating process, which helps the brain adapt to complex environments. PPI may be reduced in patients with bipolar disorder (BD). This study investigated PPI deficits in BD and pooled the effect size of PPI in patients with BD.

**Methods:**

We conducted a literature search on PPI in patients with BD from inception to July 27, 2019 in PubMed, Embase, Cochrane Library databases, and Chinese databases. No age, sex, and language restriction were set. The calculation formula was PPI = 100 - [100*((prepulse - pulse amplitude) / pulse amplitude)]. The Newcastle-Ottawa Scale (NOS) was used to assess the quality of studies.

**Results:**

Ten eligible papers were identified, of which five studies including a total of 141 euthymic patients and 132 healthy controls (HC) were included in the meta-analysis. Compared with HC, euthymic patients with BD had significantly lower PPI at the 60 ms interstimulus interval (ISI) between pulse and prepulse (*P* = 0.476, I^2^ = 0.0%, SMD = − 0.32, 95% CI = − 0.54 - -0.10). Sensitivity analysis shows no significant change in the combined effect value after removing any single study. There was no publication bias using the Egger’s test at 60 ms (*P* = 0.606). The meta-analysis of PPI at the 60 ms ISI could have significant clinical heterogeneity in mood episode state, as well as lack of data on BD I or II subtypes.

**Conclusions:**

Euthymic patients with BD show PPI deficits at the 60 ms, suggesting a deficit in the early sensory gate underlying PPI. The PPI inhibition rate at a 60 ms interval is a stable index. More research is needed in the future to confirm this outcome, and to delve deeper into the mechanisms behind deficits.

**Supplementary information:**

**Supplementary information** accompanies this paper at 10.1186/s12888-019-2271-8.

## Background

In mammals, the startle reflex is caused by a sudden and intense sensory stimulation. It is an evolved defensive reflex activity that can interrupt and interfere with ongoing cognitive and behavioral activities [1–4]. A gating mechanism can effectively inhibit the startle reflex, to ensure normal brain function. The prepulse inhibition (PPI) is the application of a weak prepulse stimulus that does not trigger a startle reflex during the first 30–500 ms before a strong stimulus. This interferes with and reduces the effects of the strong stimulus on the startle reflex [5]. PPI has good plasticity and has been widely used in various human and animal studies [6–10].

Previous studies have shown that PPI is regulated by the limbic-cortical-striatal-pallidal-thalamic (CSPT) neural circuit and the dopaminergic system [11, 12]. Further, it is found that injecting glutamatergic N-methyl-d-aspartate (NMDA) into the hippocampus can disrupt PPI by affecting the expression of Gamma-Aminobutyric Acid (GABA) neurotransmitters and the neural circuit [11, 13]. Braff et al. (1978) first found that patients diagnosed with schizophrenia had lower PPI than normal controls [14], and subsequent PPI research has focused on the schizophrenia spectrum population [15]. Moreover, PPI research with other mental disorders, including bipolar disorder (BD), obsessive-compulsive disorder, and autism spectrum disorders, has also been conducted [16–18], indicating that PPI abnormalities may be related to common psychopathological mechanisms of various disorders. PPI deficits have also been reported in first-degree relatives of patients with schizophrenia and BD, indicating that PPI may be a heritable phenotype [19, 20].

Although the neuromodulatory circuits of PPI are mainly at the brainstem level, many studies have confirmed that PPI is regulated by higher cognitive processes such as attention and emotion. Selective attention to prepulse stimulation can specifically enhance PPI in healthy subjects, while the enhancement effect in schizophrenia patients disappears due to attention deficit [21, 22]. Currently, some studies have shown that perceived spatial separation-induced PPI paradigm based on the priority effect can improve the individual recognition of prepulse sound and thus increase PPI [6]. Emotions play an important role in the selective attention and cognitive processes of people facing complex situations. For example, there can be higher PPI in response to pleasant or fearful pictures than to neutral pictures [23, 24]. Moreover, the CSPT circuit in BD patients may be impaired [25]. Deficits in this gating circuit could have adverse effects on cognitive information filtering, perhaps contributing to depressive thinking, manic thinking, and delusions.

Because of the complexity of BD, previous studies have drawn inconsistent conclusions. Some studies have found that, compared with healthy controls (HC), euthymic, or acute manic patients with BD, and first-degree relatives had significantly lower PPI levels [16, 20, 26, 27]. However, other studies found that patients with BD in the euthymic period and pediatric BD did not show PPI deficits [28–30]. There has been no systematic review or meta-analysis of PPI in patients with BD to assess the overall magnitude of these effects. The present study comprehensively retrieved the literature on PPI in patients with BD, then systematically reviewed the available data and conducted meta-analysis, comparing BD with HC.

## Methods

### Search strategy

Two authors independently searched relevant articles from the start of the database to July 27, 2019 in PubMed, Embase, the Cochrane Library databases, and the Chinese databases (VIPS, CNKI, and Wan Fang). Searches used medical subject headings (MESH), text words, and Boolean calculations. Main search terms included: bipolar disorder; bipolar affective disorder; mania; manic-depressive; manic episode; hypomania; hypomanic episode; bipolar depression; bipolar I disorder; bipolar II disorder; bipolar type I; bipolar type II; psychosis; psychoses; psychotic; prepulse inhibition; PPI; startle reflex; startle reaction; sensorimotor gating; and sensory gating. We also searched for relevant references in known quantitative papers, including non-English papers. The search strategy has been provided as supplementary material (Additional file 1). This review performed according to the Preferred Reporting Items for Systematic reviews and Meta-Analyses (PRISMA) guidelines, which provides an evidence-based minimum set of items for reporting in systematic reviews and meta-analyses. PRISMA Checklist has been included as supporting information “PRISMA checklist.doc” (Additional file 2).

### Study selection criteria

Two authors independently screened the literature by reading the titles, abstracts, and the full text. The criteria for inclusion in the meta-analysis were: (1) Presence of a HC group. When participants included people with schizophrenia, BD, and first-degree relatives, information on patients with BD was extracted. (2) The paper contained PPI data or histograms and corresponding general demographic information with a PPI formula of PPI = 100 - [100*((prepulse - pulse amplitude) /pulse amplitude)]. (3) Presence of PPI data at 60 ms interstimulus interval (ISI). If the article contains both 60 ms and 120 ms PPI, 60 ms PPI data was extracted. (4) The quality of the research was evaluated by Newcastle-Ottawa Scale (NOS) [31]. This consists of nine items divided into three domains: selection of research subjects (four items); inter-group comparability (two items); and measurement of exposure factors (three items). The star system is used for semi-quantitative evaluation of research quality. The range of NOS is from zero to nine stars. Only papers with more than 4 stars were included in this study. Review papers, abstracts from conference proceedings and case reports were excluded. If the data were incomplete, the author was contacted by email to ask for information. If the author did not give a reply, we used the GetData software to extract the data in the paper. The two authors independently intercepted data to minimize errors in this study. Finally, we converted all standard error (SE) data into standard deviation (SD) form according to the formula (SD = SE× $\sqrt{\mathrm{N}}$). Due to incomplete data, studies that cannot be converted are included in the qualitative description. When the two authors reached different conclusions about eligibility, the third author was consulted.

### Data analyses

Statistical analysis was performed using the software STATA 11.0 (Stata Corporation, USA). The heterogeneity test was evaluated using the I^2^ value. When I^2^ ≤ 50.0% and *P* ≥ 0.10, it indicated that the included studies are homogeneous, and the model of fix effect was adopted. If I^2^ is greater than 50.0% and *P* value is less than 0.10, results was considered heterogeneous, then the random effects models and sensitivity analyses were used [32]. Because the included studies used different continuous measures as outcome measures, we calculated effect sizes (Standard Mean Difference, SMD) and the standard errors of the effect sizes by the Cohen method in the metan command [33]. Galbraith graphs were plotted to test heterogeneity. Additionally, the metaninf command was used to examine the effect of individual studies on the total combined effect after removing outliers. Through the metabias command, the Egger’s test [34] was used to evaluate publication bias. The sample size, mean, SD, heterogeneity values (I^2^, p), SMD, and 95% confidence interval (CI) are shown in the forest map.

## Results

### Included studies

**Fig. 1:**
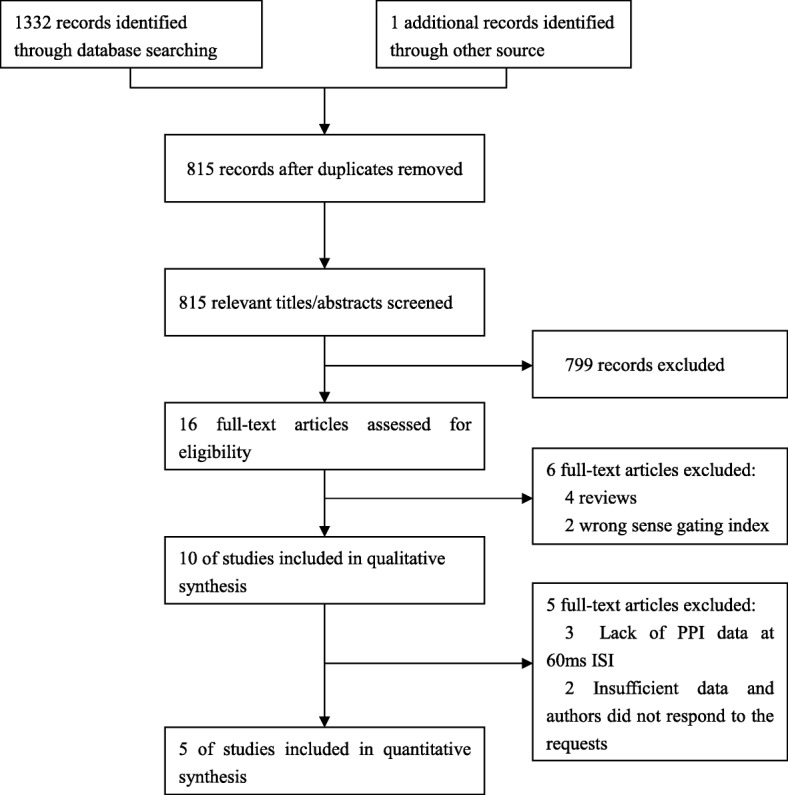
Literature search flow

Figure 1 shows the article screening process: 815 unique articles were included in the initial screening, of which all but 16 were excluded after initial screening. Six more were excluded on further screening, leaving 10 papers included in qualitative synthesis, of which 5 were excluded from meta-analyses because of the absence of general demographic data, PPI data of 60 ms ISI and the maximum and minimum values required for data conversion. Therefore, five articles were included in final quantitative analysis for 60 mm ISI. The characteristics descriptions of all studies were displayed in Table 1.

**Table 1:** Studies included in systematic review

Author (year)	Objective	Participants	Intervention	Outcome	Note
Matsuo, J. et al. 2018 [35]	To assess PPI deficits in patients with BD stratified by gender and disease status (euthymic/depressed).	106 BD (63 with depression and 43 euthymic; 26 BD I and 80 BD II) and 232 control subjects.	All individuals were evaluated using the computerized startle reflex test unit, lead Interval of prepulse-pulse is 60 ms and 120 ms.	The male BD patients with depression had significant PPI deficits, female BD patients with psychosis show lower PPI.	qualitative synthesis
QJ, Bo. et al. 2018 [36]	Using a perceived PSS-PPI paradigm to assess PPI levels in patients with BD.	30 non-manic patients with BD and 33 HC	PPI was evaluated using a modified PSS-PPI paradigm, lead Interval of prepulse-pulse is 120 ms	Patients with BD exhibited PPI deficit by using PSS-PPI paradigm. PSS-PPI deficits was significantly associated with the language domain of RBANS.	qualitative synthesis
Sanchez-Morla, E. M. et al. 2016 [16]	To assess PPI level in euthymic patients with BD.	64 euthymic patients with BD and 64 control subjects	The acoustic startle measures of PPI were performed using 60-ms and 120-ms lead interval.	Compared with HC, BD patients showed PPI deficits that is related to social cognition	qualitative/quantitative synthesis
Tamminga, C. A. et al. 2014 [37]	To assess SPEM, PPI, and ERP between schizophrenia and BD	26 psychotic bipolar I disorder and 22 HC were tested for PPI level.	Standard clinical characterization and PPI (lead Interval, 120 ms) paradigm were applied.	PPI level did not differ between psychotic bipolar I disorder and HC.	qualitative synthesis
Gogos, A. et al. 2009 [26]	To explore gender difference in PPI level in patients with BD.	29 euthymic patients with BD, and 32 HC.	Two PPI stimulus onset asynchrony levels (60, 120 ms) were assessed by 21 pulse-alone trials (115 dB) and a total of 42 prepulse-pulse trials.	Compared with controls, the male patients with BD showed reduced PPI, whereas female patients had increased PPI levels.	qualitative/quantitative synthesis
Carroll, C. A. et al. 2007 [29]	To assess PPI level in manic and mixed episode BD.	14 manic patients with BD, 21 mixed episode patients with BD and 32 HC.	The acoustic startle measures of PPI were performed using a 120-ms lead interval.	Compared to HC, mixed episode patients exhibited less PPI latency facilitation, but PPI deficits were not observed across diagnostic groups (manic, mixed, control)	qualitative synthesis
Giakoumaki, S. G. et al. 2007 [20]	To assess PPI level in remitted patients with BD and their unaffected siblings.	21 patients with BD, 19 unaffected siblings and 17 HC	The tests of acoustic startle reactivity and PPI (lead Interval, 60 ms and 120 ms) of the startle response were investigated.	Patients with BD and their unaffected siblings showed PPI deficits, and had no significant correlation with symptom and disease severity.	qualitative/quantitative synthesis
Barrett, S. L. et al. 2005 [28]	To assess PPI level in the euthymic phase of BD.	23 euthymic patients with BD, and 20 HC.	The tests of acoustic startle reactivity and PPI of the startle response were performed. Lead interval of prepulse-pulse is 60 ms and 120 ms	There was no significant PPI deficits in the euthymic phase of BD.	qualitative/quantitative synthesis
Rich, B. A. et al. 2005 [30]	To investigate PPI level in pediatric BD.	16 patients with BD (medicated, euthymic and nonpsychotic), and 13 HC.	The magnitude of startle habituation, startle-alone response, and inhibition of startle following a 60 or 120-ms prepulse were evaluated.	Pediatric BD patients did not show PPI deficits compared to healthy controls.	qualitative/quantitative synthesis
Braff, D. L. et al. 2001 [27]	To explore PPI level in BD patients with acute psychotic mania.	15 patients with BD, 16 patients with schizophrenia and 17 HC	PPI (60 ms and 120 ms interstimulus intervals) was measured using Xeye Human startle reflex system.	Compared with HC, BD patients with acute psychotic mania had PPI deficits, and the extent of deficits was not significantly different from schizophrenia	qualitative synthesis

*ISI* Interstimulus interval, *YMRS* Young Mania Rating Scale, *HAMD* Hamilton Depression Rating Scale, *PANSS* Positive and Negative Syndrome Scale, *RBANS* Repeatable Battery for the Assessment of Neuropsychological Status, *BD* Bipolar disorder, *HC* Healthy controls, *PSS-PPI* perceived spatial separation-induced prepulse inhibition, *SPEM* smooth pursuit eye movement, *ERP* auditory event-related potentials

### Meta analyses

A total of 141 euthymic patients and 132 HC were included in the meta-analysis of the 60 ms ISI. BD diagnoses in all 5 studies were according to Structured Clinical Interview for DSM Disorders-Fourth Edition (DSM-IV) criteria. The clinical characteristics and treatment of patients with BD are depicted in Table 2.

**Table 2:** Studies included in the meta-analysis

Author (year)	Sample (*N*)	Male (%)	Age (year)	Smokers (%)	Stages of disease	Psychosis History	medications	Patients clinical characteristics	Experimental paradigm	Getdata
Sanchez-Morla, E. M. et al. 2016 [16]	BD I (52)	22 (42.3)	42.6 (11.0)	18 (34.6)	Euthymic (HAMD < 7, YMRS < 6, for at least the previous 3 months)	NA	59.6% SGA, 50% Lithium, 48.1% Anticonvulsants, 30.8% Antidepressants, 30.8% Benzodiazepines	YMRS < 6 HAMD < 7	Background noise 70 dB Pulse 40 ms 118 dB Prepulse 20 ms 80 dB Interval 60 and 120 ms	No
	HC (50)	23 (46)	39.3 (10.1)	17 (34.0)						
Gogos, A. et al. 2009 [26]	BD (29)	14 (48.3)	42.9 (10.9)	9 (31.0)	Euthymic (Self-reported Euthymic)	NA	51.7% Antipsychotics,48.3% Sodium valproate, 24.1% Lithium, 37.9% Antidepressants	MRS 2.5 ± 3.7 HAMD 7.0 ± 6.3 PANSS 44 ± 9.5	Background noise 70 dB Pulse 40 ms 115 dB Prepulse 20 ms 74,78 and 86 dB Interval 60 and 120 ms	Yes
	HC (32)	16 (50)	40.5 (11.7)	1 (3.1)						
	HC (32)	15 (46.9)	30.4 (8.5)	NA						
Giakoumaki, S. G. et al. 2007 [20]	BD I (21)	11 (52.4)	32.9 (7.3)	NA	Euthymic (HAMD < 7, YMRS < 7)	*N* = 10	71.42% Antipsychotic Medication 28.57% Lithium 23.8% Valproate, 52.38% Carbamazepine	YMRS 3.29 ± 2.28 HAMD 3.43 ± 2.48, BPRS 27.00 ± 5.18, GAF 72.71 ± 9.83	Background noise 70 dB Pulse 40 ms 115 dB Prepulse 20 ms 85 dB Interval 60 and 120 ms	Yes
	HC (17)	10 (58.8)	31.6 (6.9)	NA						
Barrett, S. L. et al. 2005 [28]	BD (23)	12 (52.17)	46.4 (13.4)	10 (43.47)	Euthymic (HAMD < 8, YMRS < 20)	NA	26.09% Antipsychotics, 26.09% Sodium valproate, 78.26% Lithium 21.74% Antidepressants 8.70% Carbamazepine	YMRS 1.9 ± 3.1 HAMD 3.0 ± 2.0	Background noise 70 dB Pulse 40 ms 111 dB Prepulse 40 ms 70 and 85 dB Interval 60 or 120 ms	Yes
	HC (20)	10 (50)	42.4 (13.0)	2 (10)						
Rich, B. A. et al. 2005 [30]	BD (16)	7 (53.8)	12.7 (2.7)	NA	Euthymic (16 subjects were in euthymic period, no clear definition)	No	68.8% antipsychotics, 68.8% anticonvulsants 50.0% antidepressants, 43.8% lithium 25.0% stimulants, 25.0% sedatives	YMRS 3.71 ± 6.20 CDRS 23.14 ± 6.16, K-SADS psychosis score 2.00 ± 0.00	Background noise NA Pulse NA 104 dB Prepulse 50 ms 70 dB Interval 60 or 120 ms	Yes
	HC (13)	7 (43.8)	13.2 (2.2)	NA						

*BD* Bipolar disorder, *HC* Healthy controls, *YMRS* Young Mania Rating Scale, *GAF* Global Assessment of Functioning, *BPRS* Brief Psychiatric Rating Scale, *HAMD* Hamilton Depression Rating Scale, *MRS* Mania Rating Scale, *PANSS* Positive and Negative Syndrome Scale, *CDRS* Children’s Depression Rating Scale, *K-SADS* Kiddie-Schedule for Affective Disorders, *SGA* second-generation antipsychotics, *NA* not applicable

**Fig. 2:**
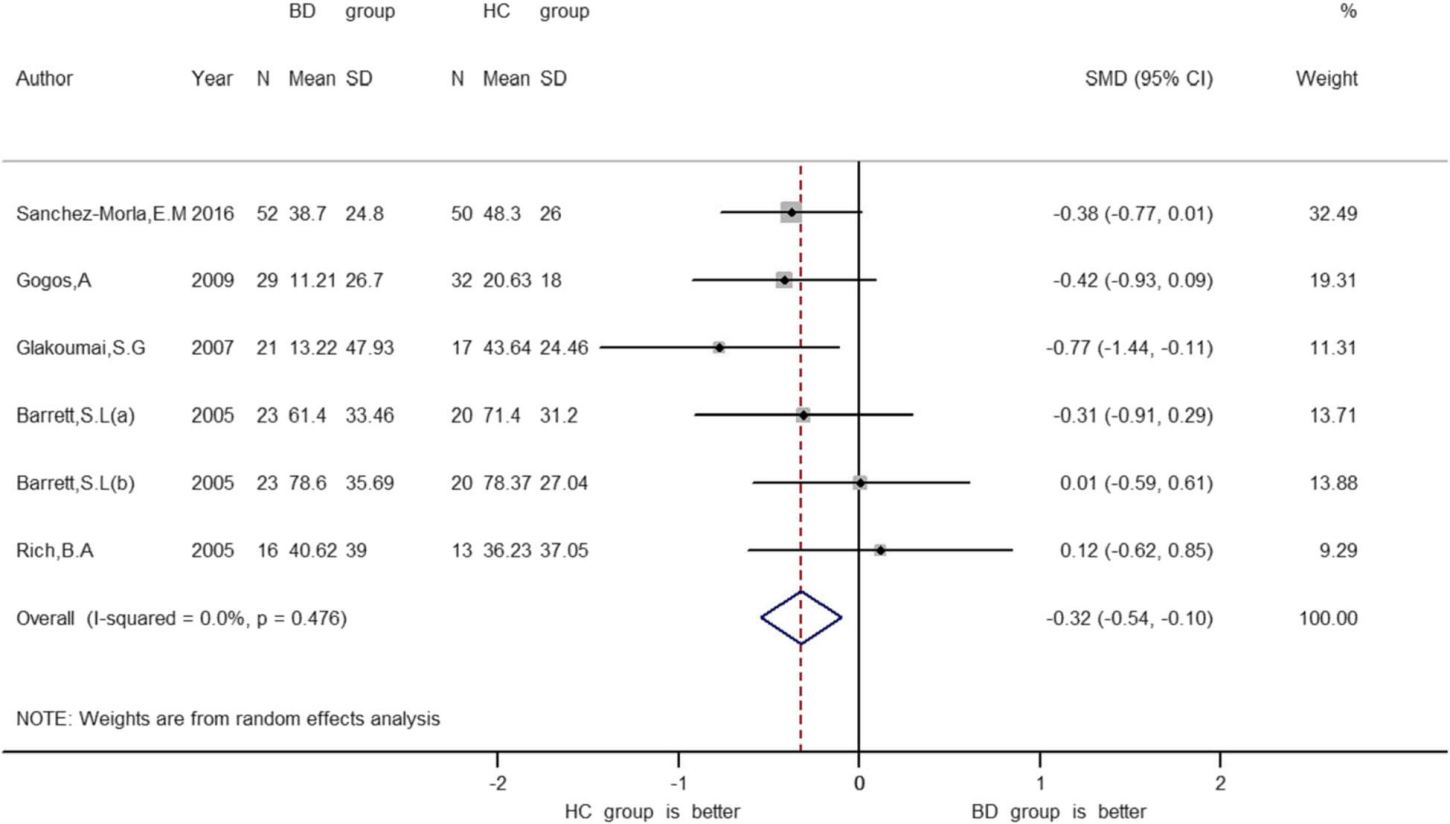
Forest plot of effect size with random effect model between BD patients and healthy controls for PPI in 60 ms interstimulus interval. The vertical solid line (0 on the abscissa scale) represents the invalid line. The dotted line represents the point estimate of all the data summarized

Furthermore, as shown in Table 2, in all five studies the PPI was calculated the same way and the mood state of patients with BD is euthymic. Therefore, we performed meta-analysis for PPI of 60 ms ISI. As can be seen in Fig. 2, there was an overall PPI difference between euthymic BD and HC at 60 ms ISI (SMD = − 0.32, 95% CI = − 0.54 – -0.10).

### Quality assessments

Using the NOS to assess the quality of the studies, all 5 papers gained 4 stars or more (Table 3).

**Table 3:** The quality assessment by NOS scale

	The Case Definition	Representativeness of the Cases	Selection of Controls	Definition of Controls	Comparability(a)	Comparability (b)	Exposure (1)	Exposure (2)	Exposure (3)^a^
Sanchez-Morla, E. M. et al. 2016	*	*		*	*	*	*	*	
Gogos, A. et al. 2009	*	*	*	*	*	*	*	*	
Giakoumaki, S. G. et al. 2007	*	*	*	*	*	*	*	*	
Barrett, S. L. et al. 2005	*	*	*	*	*	*	*	*	
Rich, B. A. et al. 2005	*			*	*	*	*	*	

*NOS* Newcastle-Ottawa Scale, ^a^When the chi-square test is greater than 0.05, it indicates that the non-response rate between the two groups has no significant significanc e[38]

Comparability: confounding factors included age, sex, race, years of education and smoking status

### Heterogeneity and sensitivity analyses

**Fig. 3:**
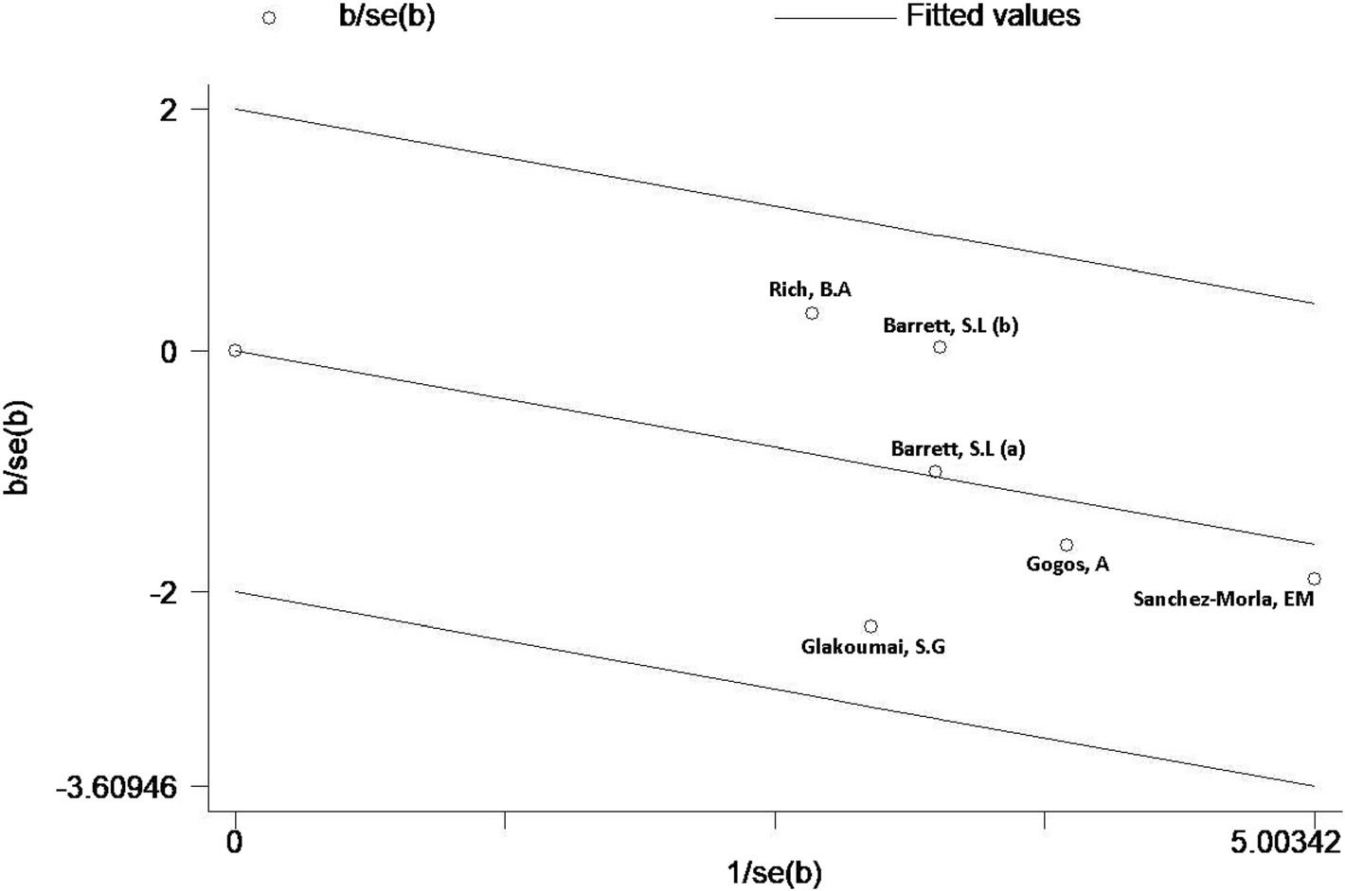
The Galbraith plot for 60 ms PPI heterogeneity. There are three diagonal lines in the figure, the middle diagonal line represents combined value of the fixed effect, and the 95% confidence interval on both sides

**Fig. 4:**
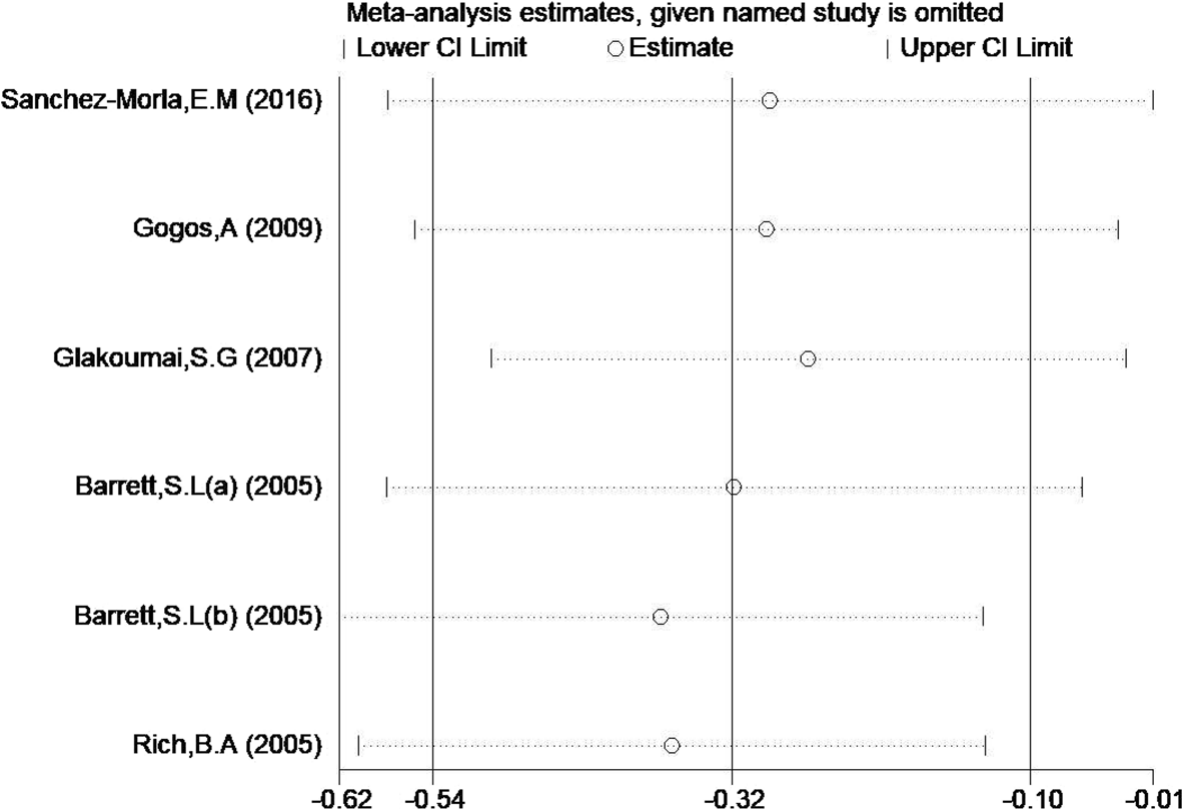
Sensitivity analysis for 60 ms PPI. The middle vertical line in the figure is a vertical line with a difference value of − 0.32, which is the total combined effect size. The points corresponding to each study represent the combined effect size of the remaining studies after deletion of the study

As shown in Fig. 2, the PPI of the 60 ms ISI had little heterogeneity (*P* = 0.476, I^2^ = 0.0%, SMD = − 0.32, 95% CI = − 0.54 - -0.10). According to the heterogeneity analysis results for 60 ms PPI, all research points fall between two regression lines (Fig. 3). Sensitivity analysis shows no significant change in the combined effect value after removing this single study (Fig. 4). Due to lack of data on BD I or II subtypes, subgroup analyses of BD type cannot be obtained.

### Publication bias

When using the Egger’s test, there was no publication bias at 60 ms (*P* = 0.606).

## Discussion

To the best of our knowledge, this is the first systematic review and meta-analysis about PPI levels in patients with BD. The main finding of this study is that euthymic patients with BD had significant PPI deficits compared with HC when the ISI between pulse and prepulse was 60 ms. In the PPI paradigm of human studies, the ISI generally range from between 30 and 240 ms. It was found that maximum PPI occurs at 60-120 ms ISI in schizophrenia patients and normal subjects [14, 39, 40]. Even if BD are treated with an mood stabilizers, atypical antipsychotics, or have a history of smoking, all of which can affect PPI, patients still have PPI deficits at 60 ms ISI. This suggests that BD is more associated with a deficit in the sensory gate at 60 ms ISI which is an insensitive interval for drugs that affect sensory gating [41–43].

The PPI at 120 ms ISI is regulated by attention and distribution in advanced cognitive function and is susceptible to drug effects [21]. A total of 10 studies explored PPI levels at 120 ms ISI in patients with BD and the conclusions are different. Four studies reported PPI defects of patients with BD and 4 studies showed no defects, other two studies explored gender differences in PPI and did not show differences between overall patients and HC. Currently, the studies include those with BD in euthymic, and mania/mixed states, as well as those with BD I or BD II, and it includes those with adults or children. The clinical heterogeneity of the 120 ms ISI in BD patients was more evident, so no meta-analysis was applied for 120 ISI. However, at present, clinical heterogeneity is difficult to explain by analyzing data. Just for the heterogeneity depend on the current analyses, further study is needed with good design.

There are multiple definitions of the euthymic period in the literature reviewed. One study suggested that euthymia was defined as score below 7 on the Hamilton Depression Rating Score (HDRS) and below 6 on the Young Mania Rating Scale (YMRS) [16]. Another used criteria of less than 8 on Hamilton Depression Rating Scale (HAMD) and less than 20 on YMRS [28]. Another included euthymic (HAMD < 7 and YMRS < 7) patients with BD [20]. Yet another study relied on self-report [26]. However, despite inconsistent criteria, by these criteria all participants were considered clinically stable at time of testing. Euthymic patients with BD had PPI deficits at 60 ms, suggesting a consistent association between BD and sensory gating deficit. This is consistent with previous studies, euthymic patients with BD still have functional deficits of sustained attention and find it difficult to ignore the irrelevant stimulus [44, 45]. At present, there is little research on PPI in the acute phase of BD and further research is needed. PPI deficits may be due to both trait and state characteristics.

Schizophrenia-related studies have shown that PPI deficits are strongly associated with positive symptoms [9, 46]. A meta-analysis of the previous P50 showed that patients with BD have sensory gate deficits, which become more serious in when exhibiting psychotic symptoms [47]. One study found that psychotic patients with BD in the manic phase have significant PPI deficits [27]. Another study of manic patients without psychotic symptoms did not find this [29]. However, Sanchez-Morla et al. support the presence of PPI deficits in stable patients, which have no association with psychotic symptoms [16]. Since PPI is found in other non-psychotic disorders and is regulated by attention, patients with BD have sustained attention deficit in different stages of the disease, so it may be a trait characteristic of BD. Unfortunately, due to insufficient PPI data, we were unable to do the relevant subgroup analysis. The relationship with the psychotic symptoms of patients with BD remains to be further studied.

PPI deficits were first demonstrated in patients with schizophrenia and have been studied primarily in patients with schizophrenia spectrum disorders [14, 15]. This meta-analysis adds to the increasing evidence that patients with BD also have impaired PPI levels, suggesting that PPI deficits are not unique to schizophrenia. However, there is overlap of symptoms and genetic communalities between BD and schizophrenia [48]. It is possible that the sensory attentional deficit assessed by PPI involves some specific neurological mechanism implicated in both disorders. BD and schizophrenia in current diagnostic systems may be different manifestations of very similar underlying brain dysfunctions and clinical phenotypes of psychiatric disorder. This is therefore a promising direction for researching ways of understanding and classifying psychiatric disorders by their biological causes, rather than by their symptom clusters.

There are some limitations of this study. PPI is affected by several types of factors. Firstly, among the literatures included in the quantitative analysis, patients in three literatures were not described the type of BD (bipolar I or II), and all the patients in the other two researches were BD I. An effective subgroup analysis cannot be performed. Secondly, according to the HAMD score, Gogos 2009 included those who were depressed assessed by HAMD [26]. The group mean is in the mild depression range, with the SD indicating that there were quite a few participants who were likely mildly depressed, but self-reported euthymic. Thirdly, PPI is affected by many factors. A number of previous studies found that the second generation of antipsychotics can improve PPI, and that mood stabilizers such as, lithium and valproate, can increase PPI [49–51]. Previously in the study of BD, most patients were prescribed drugs and had multiple drug combinations, which cannot be easily converted to equivalent dosages. PPI levels are also affected by gender, age, smoking, and other factors. Most studies suggest that male PPI levels are significantly higher than female [52, 53]. Kumari et al. suggested that the level of PPI in minors is significantly lower than in adult patients [54]. nicotine can also improve PPI levels [55]. These factors are hard to control in research and may be causes of heterogeneity. Furthermore, to carry out quantitative analysis, we use data interception software to capture data from pictures in previous literature, resulting in no deviation results between the data used and the original data.

## Conclusions

In conclusion, the current systematic review and meta-analysis suggests that BD patients show PPI deficits in the 60 ms ISI. Further research on PPI in disorders other than schizophrenia is needed, including BD during the acute phase and psychotic state, using consistent criteria for defining euthymic, acute phases, and psychotic state. More research is needed in the future to confirm this outcome, and to delve deeper into the mechanisms behind deficits.

## Supplementary information

**Additional file 1:** The search strategy. (DOCX 14 kb)

**Additional file 2:** PRISMA Checklist. (DOC 64 kb)

## Data Availability

Data are available from the first and the corresponding authors.

## Abbreviations

BD: Bipolar Disorder
CSPT: Limbic-cortical-striatal-pallidal-thalamic
DSM-IV: Structured Clinical Interview for DSM Disorders-Fourth Edition
GABA: Gamma-Aminobutyric Acid
HC: Healthy Controls
HDRS, HAMD: Hamilton Depression Rating Scale
ISI: Interstimulus Interval
MESH: Medical Subject Headings
NMDA: N-methyl-d-aspartate
NOS: Newcastle-Ottawa Scale
PPI: Prepulse Inhibition
PRISMA: Preferred Reporting Items for Systematic reviews and Meta-Analyses
YMRS: Young Mania Rating Scale
