# Supplementary material for: Prepulse inhibition in patients with bipolar disorder: a systematic review and meta-analysis
Source: BMC Psychiatry. 2019 Sep 11;19:282. doi: 10.1186/s12888-019-2271-8 (PMC6737635; doi:10.1186/s12888-019-2271-8)
Supplement: Supplementary file 1 — Additional file 1: The search strategy. (DOCX 14 kb) [file 12888_2019_2271_MOESM1_ESM.docx]

**Search strategy**

Search (((((((prepulse inhibition[Title/Abstract]) OR PPI[Title/Abstract]) OR startle reflex[Title/Abstract]) OR Startle Reaction[Title/Abstract]) OR sensory gating[Title/Abstract]) OR sensorimotor gating[Title/Abstract])) AND (((((((((((((((Bipolar Disorder[Title/Abstract]) OR Bipolar Affective Disorder[Title/Abstract]) OR Manic-Depressive[Title/Abstract]) OR Mania[Title/Abstract]) OR Manic episode[Title/Abstract]) OR Hypomania[Title/Abstract]) OR Hypomanic episode[Title/Abstract]) OR Bipolar Depression[Title/Abstract]) OR bipolar I disorder[Title/Abstract]) OR bipolar II disorder[Title/Abstract]) OR bipolar type I[Title/Abstract]) OR bipolar type II[Title/Abstract]) OR psychosis[Title/Abstract]) OR psychoses[Title/Abstract]) OR psychotic[Title/Abstract])
